# Efficient detection and classification of epigenomic changes under multiple conditions

**DOI:** 10.1111/biom.13477

**Published:** 2021-05-03

**Authors:** Pedro L. Baldoni, Naim U. Rashid, Joseph G. Ibrahim

**Affiliations:** Department of Biostatistics, University of North Carolina at Chapel Hill, Chapel Hill, North Carolina, USA

**Keywords:** ChIP-seq, differential peak call, epigenomics, hidden Markov model, mixture model

## Abstract

Epigenomics, the study of the human genome and its interactions with proteins and other cellular elements, has become of significant interest in recent years. Such interactions have been shown to regulate essential cellular functions and are associated with multiple complex diseases. Therefore, understanding how these interactions may change across conditions is central in biomedical research. Chromatin immunoprecipitation followed by massively parallel sequencing (ChIP-seq) is one of several techniques to detect local changes in epigenomic activity (peaks). However, existing methods for differential peak calling are not optimized for the diversity in ChIP-seq signal profiles, are limited to the analysis of two conditions, or cannot classify specific patterns of differential change when multiple patterns exist. To address these limitations, we present a flexible and efficient method for the detection of differential epigenomic activity across multiple conditions. We utilize data from the ENCODE Consortium and show that the presented method, epigraHMM, exhibits superior performance to current tools and it is among the fastest algorithms available, while allowing the classification of combinatorial patterns of differential epigenomic activity and the characterization of chromatin regulatory states.

## 1 INTRODUCTION

Epigenomics, the study of the genome and its interactions with proteins and cellular elements, has become of significant interest in recent years. Such interactions may regulate essential cellular functions, such as gene expression, resulting in downstream phenotypic impact (Kim et al., 2018). Hence, the interrogation of how these interactions may change across conditions, such as cell types or treatments, is of marked interest in biomedical research. Several landmark articles have identified genomic regions of changing (differential) epigenomic activity between conditions as drivers of cell differentiation (Creyghton et al., 2010) and a number of human diseases (Portela and Esteller, 2010). Within differential regions, delineating specific patterns of change across conditions is also of interest, for example, classifying the gain-of-activity or loss-of-activity in genomic loci due to treatment (Clouaire et al., 2014).

To quantify local epigenomic activity, a common high-throughput assay is chromatin immunoprecipitation followed by massively parallel sequencing (ChIP-seq). ChIP-seq begins with cross-linking DNA and proteins within chromatin structures, which are then fragmented by sonication. DNA fragments bound to the protein of interest are isolated by immunoprecipitation and sequenced via high-throughput sequencing to generate short reads pertaining to the original fragments. Sequences are then mapped onto a reference genome to determine their likely locations of origin. Genomic coordinates containing a high density of mapped reads (enrichment regions, peaks) indicate likely locations of protein–DNA interaction sites, while other regions are referred to as background regions. The read density is often summarized by counting reads mapped onto non-overlapping windows of fixed length (window read counts), forming the basis for downstream analyses. Across multiple conditions, regions exhibiting enrichment in at least one condition, but not across all conditions, indicate the presence of differential activity pertaining to the protein–DNA interaction of interest.

To date, many differential peak callers (DPCs) have been proposed. However, several challenges affect their ability to accurately detect regions of differential activity from the wide range of experiments (Section 2). First, differential regions may be both short or broad, causing difficulty for methods optimized for a particular type of peak profile (Stark and Brown, 2011; Chen et al., 2015). Second, pooling experimental replicates often leads to lower specificity in comparison to joint modeling samples from each condition (Song and Smith, 2011). Third, ChIP-seq data is often subject to complex biases that vary across the genome, as differences in local read enrichment may depend on quantities such as local read abundance or GC content (Teng and Irizarry, 2017). DPCs that ignore such effects, or rely on global scaling factors or control subtraction methods (Shen et al., 2013; Allhoff et al., 2016), may be subject to spurious differences due to the lack of nonlinear normalization methods (Lun and Smyth, 2015). In ChIP-seq data with broad peaks, a recent comparison showed that methods often detect a large number of either short or false positive peaks (Steinhauser et al., 2016). Moreover, only few methods can detect or classify differential combinatorial patterns across any number of conditions (Stark and Brown, 2011; Chen et al., 2015; Lun and Smyth, 2015).

**FIGURE 1:**
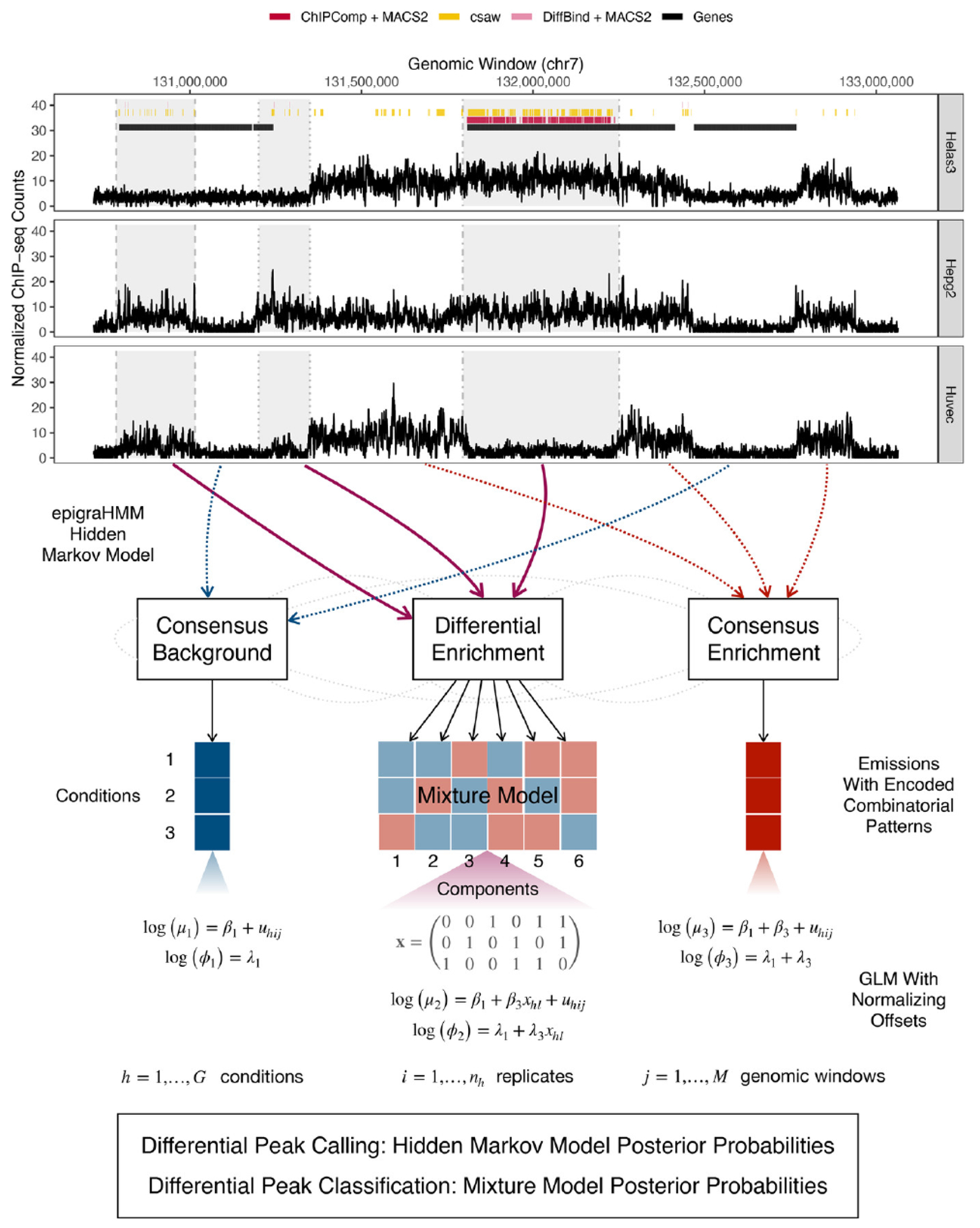
At the top, differential peak calls from current methods (under false discovery rate control of 0.05) for the histone modification H3K27me3 among cell lines Helas3, Hepg2, and Huvec. Shaded regions indicate observed differential enrichment, and each vertical line type bordering each region represents a different combinatorial pattern of enrichment across cell lines. Optimal methods would call broad peaks inside shaded regions and no peaks outside them. At the bottom, general overview of the presented method for simultaneous detection and classification of broad and short differential regions of enrichment. This figure appears in color in the electronic version of this article, and any mention of color refers to that version.

Here, we present epigraHMM (Figure 1), an efficient and flexible statistical method to identify differential regions of enrichment from epigenomic experiments with diverse signal profiles. We overcome the limitations of current DPCs with three major features. First, it uses a hidden Markov model (HMM) to account for the diversity in differential enrichment from broad and short ChIP-seq data collected under multi-replicate, multi-condition settings. Second, it models specific combinatorial patterns of enrichment via a finite mixture model emission distribution within the HMM differential state. Each mixture component pertains to a particular differential combinatorial pattern originated by the presence or absence of local enrichment across conditions, where a generalized linear model (GLM) encodes specific differential combinatorial patterns while accounting for sample- and window-specific normalizing offsets. Third, it simultaneously detects and classifies epigenomic changes under three or more conditions, a novelty not yet available in any other DPC algorithm. epigraHMM offers benefits over current HMM algorithms (Song and Smith, 2011; Allhoff et al., 2016) with an embedded GLM mixture model that allows the modeling of covariates of interest, the inclusion of normalizing offsets for nonlinear biases (such as GC-content bias), and a fast estimation scheme via rejection-controlled EM algorithm (RCEM; Ma et al. (2006)).

## 2 DATA

Histones are proteins that condense the DNA in eukaryotic cells into units called nucleosomes and, when enzymatically modified, mediate changes in local DNA packaging and chromatin accessibility. Hence, cellular processes such as gene transcription, gene silencing, and DNA repair are also affected. Proteins that interact with DNA and alter its functional properties are often referred to as epigenomic marks. The histone modifications H3K36me3 and H3K27me3 are examples of marks that associate with genomic loci containing transcribed and repressed genes (Liu et al., 2016), respectively, while exhibiting broad enrichment profiles. The enhancer of zeste homolog 2 (EZH2), a component of the complex PRC2 that catalyzes the methylation of H3K27me3 (Margueron and Reinberg, 2011), is another example of a protein characterized by broad enrichment domains and co-occurs with the activity of H3K27me3. Conversely, short peaks from histone modifications H3K27ac and H3K4me3 are usually deposited on the promoter regions of transcribed genes and several studies have associated their role with gene transcription (Creyghton et al., 2010). Similarly, the transcription factor CTCF is a protein that binds to short DNA motifs and is responsible for cellular processes that include the regulation of the chromatin 3D structure (Shukla et al., 2011).

Using ChIP-seq data pertaining to histone modifications H3K27me3 and H3K36me3, and to EZH2 from the ENCODE Consortium (Landt et al., 2012), we find that current DPCs have difficulty in accurately detecting broad regions of differential enrichment between several common cell lines (Figure 1). In line with previous findings (Steinhauser et al., 2016), we observe that even current DPCs designed for broad data (Song and Smith, 2011; Allhoff et al., 2016) tend to either call overly fragmented differential peaks or call regions exhibiting no difference in experimental signal between conditions as differential (Sections 4.2 and 5.1). Methods that rely on candidate peaks may also exhibit a compromised performance due to the limitations of single-sample peak callers in broad data (Stark and Brown, 2011; Chen et al., 2015). Moreover, most DPCs restrict their application to the analysis of two experimental conditions. For DPCs tailored for the analysis of three or more conditions, the classification of specific differential combinatorial patterns across conditions (or across various epigenomic processes) is still an open problem. The classification of such patterns would allow researchers to quantify treatment responses on the epigenomic level or identify sets of processes working together to regulate local chromatin state (Clouaire et al., 2014).

We assessed the performance of our model on ChIP-seq experiments characterized by broad peaks (H3K36me3, H3K27me3, and EZH2) and short peaks (H3K27ac, H3K4me3, and CTCF). In simulated and in real data from the ENCODE Consortium (Sections 4 and 5), our model addresses the issues of the current peak callers in broad data, while being flexible for short peaks and comparable to the fastest DPCs regarding the computation time. We show that our method can also be utilized for genomic regulatory state segmentation when studying multiple types of epigenomic processes from a single condition or cell line (Section 5.3). Online Appendices A1–A4 present data accession codes, data pre-processing steps, code implementing the method, and scripts to replicate the presented results, respectively.

## 3 METHODS

### 3.1 Statistical model

Let $Y_{\text{hij}}$ denote the random variable pertaining to the read count for genomic window j from sample i of condition h, where $j=1,\ldots ,M,i=1,\ldots ,n_{h},h=1,\ldots ,G$, and let $y_{\mathrm{hij}}$ be the observed count. Here, $n_{h}$ is the number of samples in condition h and $N=\sum_{h=1}^{G}n_{h}$ is the total number of samples across the G conditions. At the j th window, let $y_{..j}={\left(y_{11j},\ldots ,y_{Gn_{G}j}\right)}^{\prime }$ denote the N×1 vector of window read counts across all samples and conditions, and let $y=\left(y_{..1},\ldots ,y_{..M}\right)$ denote the corresponding N×M matrix of window read counts spanning all windows, samples, and conditions. We assume that each window belongs to one of three possible hidden states: consensus background, differential, and consensus enrichment. Windows exhibiting low (high) enrichment across all conditions will be modeled by an emission distribution pertaining to the consensus background (enrichment) state. Windows exhibiting enrichment under at least one condition, but not all conditions, will be modeled by an emission distribution pertaining to the differential state. If G conditions are of interest, there are $L=2^{G}-2$ possible differential combinatorial patterns of enrichment and background across conditions at a given window. The emission distribution pertainingto the differential state models all L possible differential combinatorial patterns via a mixture model with mixture proportions $\delta ={\left(\delta_{1},\ldots ,\delta_{L}\right)}^{\prime }$, such that $\sum_{l=1}^{L}\delta_{l}=1$.

We assume a single latent discrete time stationary Markov chain $Z={\left\{Z_{j}\right\}}_{j=1}^{M},Z_{j}\in \{1,2,3\}$, with state-to-state transition probabilities $\gamma ={\left(\gamma_{11},\gamma_{12},\ldots ,\gamma_{33}\right)}^{\prime }$ and initial probabilities $\pi ={\left(\pi_{1},\pi_{2},\pi_{3}\right)}^{\prime }$, such that $\sum_{s=1}^{3}\gamma_{rs}=$ 1 and $\sum_{s=1}^{3}\pi_{s}=1$ for r∈{1,2,3}. Let $f_{r}\left(y_{..j}|\psi_{r}\right)$ denote the emission distribution corresponding to the r th hidden state, $\Psi ={\left(\pi^{\prime },\gamma^{\prime },\delta^{\prime },\psi^{\prime }\right)}^{\prime }$ denote the vector of all model parameters, $\psi ={\left(\psi_{1}^{\prime },\psi_{2}^{\prime },\psi_{3}^{\prime }\right)}^{\prime }$ denote each state’s set of emission distribution-specific parameters, and 𝒵 denote the set of $3^{M}$ possible state paths of length M. The likelihood function of the proposed HMM may be written as

(1) $$f(y|x;\Psi )=\underset{Z\in 𝒵}{\sum }\{\overset{3}{\underset{r=1}{\prod }}\pi_{r}^{I\left(Z_{1}=r\right)}\times \left(\overset{M}{\underset{j=2}{\prod }}\overset{3}{\underset{r=1}{\prod }}\overset{3}{\underset{s=1}{\prod }}\gamma_{rs}^{I\left(Z_{j-1}=r,Z_{j}=s\right)}\right)\;\times (\overset{M}{\underset{j=1}{\prod }}f_{1}{\left(y_{..j}|\psi_{1}\right)}^{I\left(Z_{j}=1\right)}f_{2}{\left(y_{..j}|x;\delta ,\psi_{2}\right)}^{I\left(Z_{j}=2\right)}\;\times f_{3}{\left(y_{..j}|\psi_{3}\right)}^{I\left(Z_{j}=3\right)})\}.$$

Here, x is a fixed G×L design matrix enumerating each of the L possible differential combinatorial patterns in terms of the presence or absence of enrichment across each of the G conditions, only in the emission distribution of the differential state. We assume that read counts pertaining to genomic windows from the consensus background (r=1) and consensus enrichment ( r=3 ) states follow a negative binomial (NB) distribution with state-specific parameters $\psi_{r}={\left(\mu_{\left(r,hij\right)},\phi_{r}\right)}^{\prime }$, with mean $\mu_{\left(r,hij\right)}$ and variance $\mu_{\left(r,hij\right)}\left(1+\mu_{\left(r,hij\right)}/\phi_{r}\right)$. Conditional on the HMM state, we assume independence of read counts across samples and write the emission distribution of the consensus background and enrichment states as

(2) $$f_{r}\left(y_{..j}|\psi_{r}\right)=\overset{G}{\underset{h=1}{\prod }}\overset{n_{h}}{\underset{i=1}{\prod }}\frac{\text{Γ}\left(y_{\mathrm{hij}}+\phi_{r}\right)}{y_{\mathrm{hij}}!\text{Γ}\left(\phi_{r}\right)}{\left(\frac{\phi_{r}}{\mu_{\left(r,hij\right)}+\phi_{r}}\right)}^{\phi_{r}}\;\times {\left(\frac{\mu_{\left(r,hij\right)}}{\mu_{\left(r,hij\right)}+\phi_{r}}\right)}^{y_{\mathrm{hij}}},\;r\in \{1,3\},$$

such that $log\left(\mu_{\left(1,hij\right)}\right)=\beta_{1}+u_{\mathrm{hij}},log\left(\phi_{1}\right)=\lambda_{1}$, $log\left(\mu_{\left(3,hij\right)}\right)=\beta_{1}+\beta_{3}+u_{\mathrm{hij}},$ and $log\left(\phi_{3}\right)=\lambda_{1}+\lambda_{3}$. The offset $u_{\mathrm{hij}}$ may adjust for technical or biological artifacts such as the GC-content bias and allows the nonlinear normalization of read counts across genomic windows, replicates, and conditions (online Appendix B1; Teng and Irizarry (2017)).

In the differential state ( r=2 ), read counts are modeled by a L-component finite mixture model with mixture components that follow a NB distribution, where each component corresponds to a particular differential combinatorial pattern. To define these patterns, consider sets $S_{1},\ldots ,S_{L}$ delineating the subset of the G conditions that are enriched in each of the L differential combinatorial patterns. For instance, if G=3, the sets $S_{1}=\{1\},S_{2}=\{2\},S_{3}=\{3\},S_{4}=\{1,2\},S_{5}=\{1,3\}$, and $S_{6}=\{2,3\}$ define the six possible differential combinatorial patterns of enrichment and background across three conditions (e.g., $S_{6}$ denotes enrichment in conditions 2 and 3 and background in condition 1). The presence or absence of enrichment in each of the L sets is encoded into each column of $x=\left(x_{1},\ldots ,x_{L}\right)$, such that $x_{l}={\left(x_{1l},\ldots ,x_{Gl}\right)}^{\prime }$, and $x_{hl}=I\left(h\in S_{l}\right)$ for l=1,…,L and h=1,…,G. That is, $x_{l}$ is the G×1 vector of binary indicator variables denoting which subset of conditions are enriched in pattern (mixture component) l. Let $\psi_{2}$ and $\psi_{\left(2,l\right)}$ denote the parameter vectors pertaining to the differential state and to the l th mixture component, respectively. Conditional on the differential state, we assume independence of read counts across samples and write the finite mixture model emission distribution as

(3) $$f_{2}\left(y_{..j}|x;\delta ,\psi_{2}\right)=\overset{L}{\underset{l=1}{\sum }}\delta_{l}\overset{G}{\underset{h=1}{\prod }}\overset{n_{h}}{\underset{i=1}{\prod }}\frac{\text{Γ}\left(y_{\mathrm{hij}}+\phi_{\left(2,\mathrm{lh}\right)}\right)}{y_{\mathrm{hij}}!\text{Γ}\left(\phi_{\left(2,\mathrm{lh}\right)}\right)}\;\times {\left(\frac{\phi_{\left(2,\mathrm{lh}\right)}}{\mu_{\left(2,\mathrm{lhij}\right)}+\phi_{\left(2,\mathrm{lh}\right)}}\right)}^{\phi_{\left(2,\mathrm{lh}\right)}}\;\times {\left(\frac{\mu_{\left(2,\mathrm{lhij}\right)}}{\mu_{\left(2,\mathrm{lhij}\right)}+\phi_{\left(2,\mathrm{lh}\right)}}\right)}^{y_{\mathrm{hij}}},$$

where $\mu_{\left(2,\mathrm{lhij}\right)}$ and $\phi_{\left(2,\mathrm{lh}\right)}$ are the mean and dispersion, respectively, pertaining to read counts originating from window j and sample i in condition h from the mixture component l. We assume that $log\left(\mu_{\left(2,\mathrm{lhij}\right)}\right)=\beta_{1}+\beta_{3}x_{hl}+u_{\mathrm{hij}}$ and $log\left(\phi_{\left(2,\mathrm{lh}\right)}\right)=\lambda_{1}+\lambda_{3}x_{hl}$. That is, in the mixture component l, we utilize the same consensus background (consensus enriched) parametrization from (2) in all conditions that $x_{l}$ specifies to be background (enriched) in the l th differential combinatorial pattern. Such a parametrization ensures that windows exhibiting differential enrichment across conditions share common means and dispersions between the consensus background enrichment states, an assumption that increases computational efficiency. Utilizing a mixture model as the differential state emission distribution avoids the computational burden that would come from assuming separate hidden states for each of the L differential combinatorial patterns. We evaluate the strength of these assumptions through multiple simulations and a real data benchmarking analysis in Sections 4 and 5.

### 3.2 Estimation

Here, consider a set of latent variables $W={\left(W_{1}^{\prime },\ldots ,W_{M}^{\prime }\right)}^{\prime }$, such that $W_{j}={\left(W_{j1},\ldots ,W_{jL}\right)}^{\prime }$ for j=1,…,M. We assume that W is a sequence of independent random vectors such that $W_{j}|\left(Z_{j}=2\right)∼Multinomial(1,\delta )$ and $W_{j}|\left(Z_{j}=r\right)=0$ with probability 1 if r={1,3}. The data generating mechanism can be then interpreted as read counts pertaining to window j being sampled from $f_{\left(2,l\right)}$, given $\psi_{\left(2,l\right)}$ and $x_{l}$, when $Z_{j}=2$ (differential state) and $W_{jl}=1$ (lth differential combinatorial pattern). Denoting 𝒲 as the set of $L^{M}$ possible combinations of latent vectors W, the likelihood function of the observed data (1) can be rewritten as

$$f(y|x;\Psi )=\underset{Z\in 𝒵}{\sum }\underset{W\in 𝒲}{\sum }\{\left[\overset{3}{\underset{r=1}{\prod }}\pi_{r}^{I\left(Z_{1}=r\right)}\overset{M}{\underset{j=2}{\prod }}\overset{3}{\underset{r=1}{\prod }}\overset{3}{\underset{s=1}{\prod }}\gamma_{rs}^{I\left(Z_{j-1}=r,Z_{j}=s\right)}\right]\;\times \overset{M}{\underset{j=1}{\prod }}\overset{L}{\underset{l=1}{\prod }}\delta_{l}^{W_{jl}I\left(Z_{j}=2\right)}[\overset{M}{\underset{j=1}{\prod }}f_{1}{\left(y_{..j}|\psi_{1}\right)}^{I\left(Z_{j}=1\right)}\;\times {\left(\overset{L}{\underset{l=1}{\prod }}f_{\left(2,l\right)}{\left(y_{..j}|x_{l};\psi_{\left(2,l\right)}\right)}^{W_{jl}}\right)}^{I\left(Z_{j}=2\right)}\;\times f_{3}{\left(y_{..j}|\psi_{3}\right)}^{I\left(Z_{j}=3\right)}]\},$$

where $f_{\left(2,l\right)}\left(y_{..j}|x_{l};\psi_{\left(2,l\right)}\right)$ is defined as in (3). In the t th iteration of the EM algorithm, the Q function of the complete data log-likelihood can be written as

(5) $$Q\left(\Psi |\Psi^{\left(t\right)}\right)=E_{Z}\left(E_{W|Z}\left(\text{log}(f(y,W,Z|x;\Psi ))|y,x;\Psi^{\left(t\right)}\right)|y,x;\Psi^{\left(t\right)}\right),\;=Q_{0}\left(\pi ,\gamma |\Psi^{\left(t\right)}\right)+Q_{1}\left(\psi_{1}|\Psi^{\left(t\right)}\right)+Q_{2}\left(\delta ,\psi_{2}|\Psi^{\left(t\right)}\right)+Q_{3}\left(\psi_{3}|\Psi^{\left(t\right)}\right),$$

where $Q_{0}\left(\pi ,\gamma |\Psi^{\left(t\right)}\right),Q_{1}\left(\psi_{1}|\Psi^{\left(t\right)}\right),Q_{2}\left(\delta ,\psi_{2}|\Psi^{\left(t\right)}\right)$, and $Q_{3}\left(\psi_{3}|\Psi^{\left(t\right)}\right)$ are defined in the online Appendix B4. In the E-step of the EM algorithm, we compute the posterior probabilities from (5). The quantities $Pr\left(Z_{j}=r|y,x;\Psi^{\left(t\right)}\right)$ and $Pr\left(Z_{j-1}=r,Z_{j}=s|y,x;\Psi^{\left(t\right)}\right)$ can be calculated through the Forward-Backward algorithm (online Appendix B4) and $Pr\left(W_{jl}=1|Z_{j}=2,y_{..j},x;\Psi^{\left(t\right)}\right)=f_{\left(2,l\right)}\left(y_{..j}|x_{l};\psi_{\left(2,l\right)}^{\left(t\right)}\right)\delta_{l}^{\left(t\right)}/\sum_{k=1}^{L}f_{\left(2,k\right)}\left(y_{..j}|x_{k};\psi_{\left(2,k\right)}^{\left(t\right)}\right)\delta_{k}^{\left(t\right)}$ for *l* = 1, …, *L*.

The Q function is maximized with respect to the parameters $\Psi ={\left(\pi^{\prime },\gamma^{\prime },\delta^{\prime },\beta_{1},\beta_{3},\lambda_{1},\lambda_{3}\right)}^{\prime }$ during the M-step of the algorithm. Estimates of the initial and transition probabilities can be directly calculated as ${\overset{ˆ}{\pi }}_{r}^{\left(t+1\right)}=Pr\left(Z_{1}=r|y,x;\Psi^{\left(t\right)}\right)$ and ${\overset{ˆ}{\gamma }}_{rs}^{\left(t+1\right)}=\sum_{j=2}^{M}Pr\left(Z_{j-1}=r,Z_{j}=s|y,x;\Psi^{\left(t\right)}\right)/\sum_{j=2}^{M}Pr\left(Z_{j-1}=\right.\left.r|y,x;\Psi^{\left(t\right)}\right)$, respectively, restricted to $\sum_{r=1}^{3}{\overset{ˆ}{\pi }}_{r}^{\left(t+1\right)}=1$ and $\sum_{s=1}^{3}{\overset{ˆ}{\gamma }}_{rs}^{\left(t+1\right)}=1$, for r∈{1,2,3}. We perform conditional maximizations to estimate the remaining parameters ${\left(\delta^{\prime },\beta_{1},\beta_{3},\lambda_{1},\lambda_{3}\right)}^{\prime }$. First, mixture proportions can be estimated as ${\overset{ˆ}{\delta }}_{l}^{\left(t+1\right)}=\sum_{j=1}^{M}Pr\left(Z_{j}=2|y,x;\Psi^{\left(t\right)}\right)Pr\left(W_{jl}=\right.\left.1|Z_{j}=2,y_{..j},x;\Psi^{\left(t\right)}\right)/\sum_{j=1}^{M}Pr\left(Z_{j}=2|y,x;\Psi^{\left(t\right)}\right)$. Then, estimates of ${\left(\beta_{1},\beta_{3},\lambda_{1},\lambda_{3}\right)}^{\prime }$ are obtained in a similar fashion to the parameter estimation of a weighted NB regression model (online Appendix B4).

The EM algorithm iterates until the maximum absolute relative change in the parameter estimates three iterations apart is less than $10^{-3}$ for three consecutive iterations. Here, we use an RCEM algorithm with threshold 0.05, which substantially reduces the dimensionality of the data during the M-step by randomly assigning a zero posterior probability to genomic windows unlikely to belong to each of the HMM states. Moreover, our estimation scheme allows genomic windows with equal distribution of counts across replicates and conditions to have their posterior probability aggregated during the M-step of the algorithm, which leads to a fast gradient-based optimization. Upon convergence, HMM posterior probabilities can be used to segment the genome into consensus background, differential, or consensus enrichment windows. Approaches that control the total false discovery rate (FDR) via posterior probabilities (Efron et al., 2001) or that estimate the most likely sequence of hidden states can be used. Let ${\overset{ˆ}{\rho }}_{j2}=Pr\left(Z_{j}=2|y,x;\overset{ˆ}{\Psi }\right)$ denote the estimated posterior probability that the j th genomic window belongs to the differential HMM state, j=1,…,M. A posterior probability cutoff α is then chosen by controlling the total FDR $\sum_{j=1}^{M}\left(1-{\overset{ˆ}{\rho }}_{j2}\right)I\left({\overset{ˆ}{\rho }}_{j2}\geq 1-\alpha \right)/\sum_{j=1}^{M}I\left({\overset{ˆ}{\rho }}_{j2}\geq 1-\alpha \right)$, where I(⋅) is an indicator function. Differential peaks are formed by merging adjacent windows that either meet a nominal FDR level for the differential HMM state or belong to the same predicted state (online Appendix B3). Under this set up, we are able not only to detect differential enrichment regions across multiple conditions, but we can also classify various differential combinatorial patterns of enrichment within broad and short domains with mixture model posterior probabilities.

The estimation scheme is robust to situations where certain differential combinatorial patterns of enrichment are rare (Figure 5), which results from the fact that ChIP-seq experiments often provide enough data to estimate the parameters ${\left(\beta_{1},\beta_{3},\lambda_{1},\lambda_{3}\right)}^{\prime }$ shared across all L mixture components and HMM states. If pruning differential combinatorial patterns of the mixture model is of interest, the optimal number of mixture components $L^{*},L^{*}<L$, can be selected via the Bayesian information criterion (BIC) for HMMs. We observed that selecting the optimal $L^{*}$ and combinatorial patterns via BIC agrees with pruning of rare differential combinatorial patterns that one would not expect to observe biologically (online Appendix B2). In addition, the current implementation of epigraHMM allows for the adjustment for external covariates, such as GC-content and input controls, via model offsets (online Appendix A3). In real data analyses, however, we did not observe a significant influence of input controls on the sensitivity and specificity of DPCs, a fact that has also been noted by others (Lun and Smyth, 2015).

## 4 | SIMULATION STUDIES

We evaluated epigraHMM in two simulation studies. First, we simulated read count-based data to assess the precision of the estimation scheme, the performance of differential peak detection, and the accuracy of the classification of differential combinatorial patterns (Section 4.1). Next, we utilized the simulation pipeline from Lun and Smyth (2015) to simulate ChIP-seq reads from experiments with broad differential peaks (Section 4.2). The aim of the second simulation study was to compare epigraHMM with other DPCs in a more realistic scenario with broad peaks, while avoiding the choice of a parametric model for the data.

### 4.1 | Read count simulation

Read counts were simulated under scenarios that varied regarding the type of histone modification mark (H3K36me3 and H3K27me3), number of windows (*M*, 10^5^ , 5 × 10^5^, and 10^6^ windows), number of conditions (*G*, 2, 3, and 4), and number of replicates per condition (*n*, 1, 2, and 4). We further assessed epigraHMM under different signal-to-noise ratio (SNR) levels, here defined as the ratio between the means of consensus enrichment and background emission distributions, by decreasing the mean ratio while maintaining the mean–variance relationship. Model parameters were estimated from ENCODE data (online Appendix C1). Simulated counts followed a NB distribution and were generated using a first-order Markov chain with 2*^G^* states, representing every combination of background and enrichment across *G* conditions. We assessed whether epigraHMM was able to assign all 2*^G^* – 2 differential states to the differential HMM state, while precisely estimating model parameters and accurately classifying differential combinatorial patterns.

Table 1 presents results relative to the H3K27me3 simulation scenario with 10^6^ genomic windows and ENCODE-estimated SNR (mean ratio of 3.21; see the online Appendix C1 for additional details). Depending on the number of conditions, the relative bias and the range of the reported percentiles tended to decrease as more replicates were included in the analyses. This effect was more pronounced when estimating *β*_3_ and *λ*_3_ in scenarios with four conditions, which highlights the importance of experimental replicates to achieve precise parameter estimates. Overall, the proposed estimation scheme led to precise parameter estimates and was robust to a data generating mechanism that was different from the one assumed by the proposed model. No significant differences regarding the relative bias of parameter estimates were observed across simulations under different number of genomic windows.

### 4.2 | Sequencing read simulation

Here, we compared epigraHMM with the widely used DPCs ChIPComp, csaw, DiffBind, diffReps, RSEG, and THOR, a list that covers a variety of algorithms for both broad and short ChIP-seq enrichment profiles, on data simulated with the pipeline from Lun and Smyth (2015) without a particular model assumption. Sequencing reads from ChIP-seq experiments were generated for two conditions and two replicates per condition. For the two-step DPCs ChIPComp and DiffBind, we followed Lun and Smyth (2015) and called peaks in advance using HOMER (Heinz et al., 2010), which were then used as input in the respective software for differential call. A hundred simulated data sets were generated and peaks were called under multiple nominal FDR thresholds. For epigraHMM and RSEG, window-based posterior probabilities were used to control the total FDR (Section 3.2).

**FIGURE 2:**
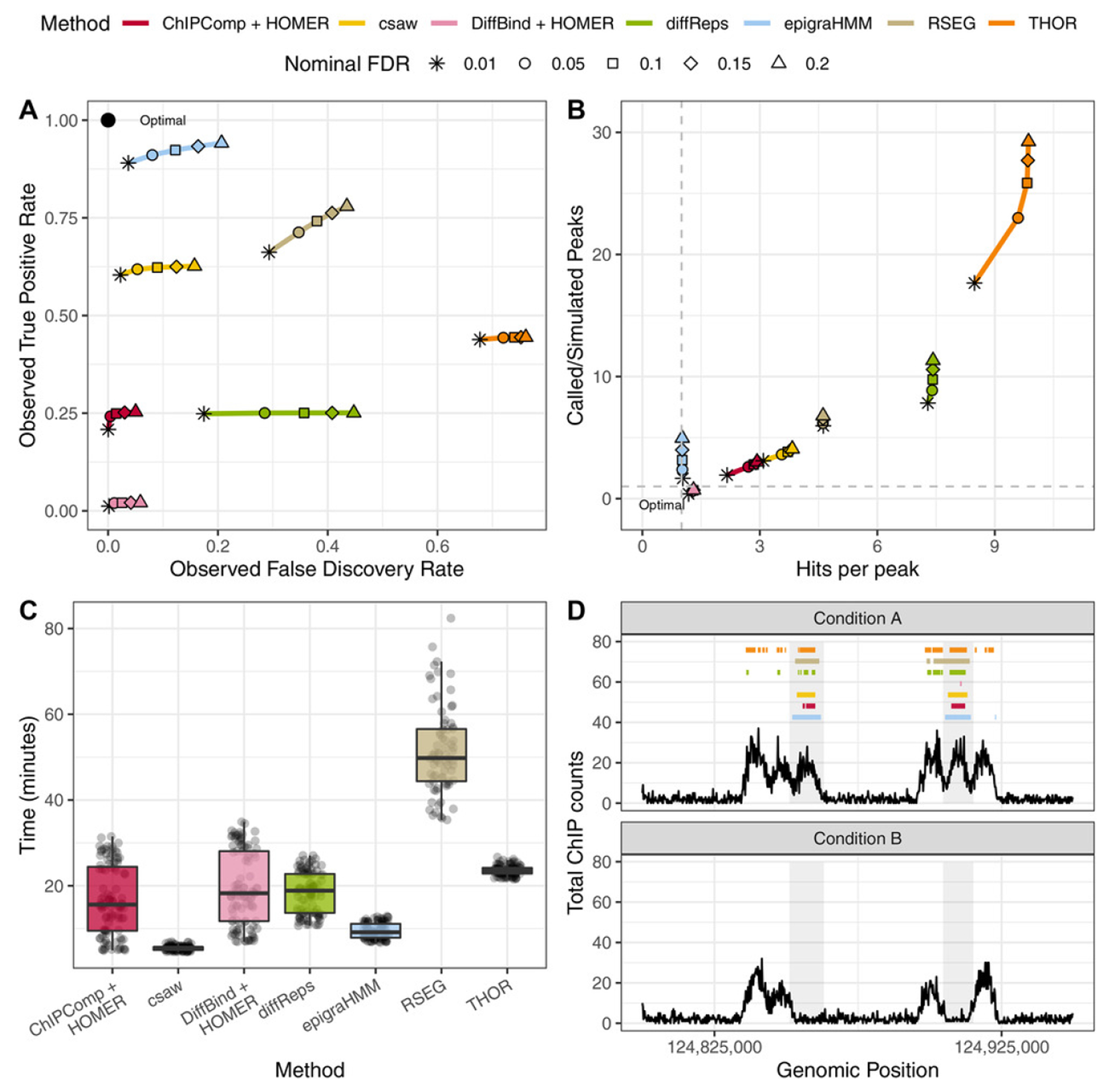
Sequencing read-based simulation from the csaw pipeline. (A) average observed sensitivity and FDR for various methods. (B) Scatter plot of average ratio of called and simulated peaks (*y*-axis) and number of called peaks intersecting true differential regions (*x*-axis). (C) Box plot of computing time (in minutes) for various algorithms. (D) An example of DPCs under a nominal FDR control of 0.05. Shaded areas indicate true differential peaks. This figure appears in color in the electronic version of this article, and any mention of color refers to that version.

Overall, epigraHMM showed the highest observed sensitivity among all DPCs while maintaining an observed FDR close to the nominal value across a wide range of nominal FDR levels (Figure 2A). Here, the observed FDR is the proportion of windows incorrectly called as differential out of the total number of windows called as differential. Due to the excessive number of false positive DPCs, diffReps, RSEG, and THOR showed higher observed FDR levels than the nominal FDR values (Figure 2D). Overall, diffReps and THOR called an excessive number of short and discontiguous peaks while RSEG called regions that were usually wider than the simulated differential peaks and did not correspond to simulated differential enrichment (Figure 2B). Regarding the computation time, epigraHMM was among the fastest DPCs due to the efficient RCEM implementation (Figure 2C). Other HMM-based algorithms, RSEG and THOR, appeared to be the most computationally intensive and required longer amounts of time to analyze the data. In general, epigraHMM was able to consistently cover most of the true differential regions with broad peaks while exhibiting a limited number of false discoveries (Figure 2D; online Appendix C2). Results shown for RSEG do not include 27 cases in which the algorithm failed to analyze the data due to internal errors or called the entire genome as differential. Similar issues of RSEG have been reported by others (Starmer and Magnuson, 2016).

## 5 | APPLICATION TO ENCODE DATA

We applied epigraHMM on ChIP-seq data from the ENCODE Consortium to detect differential peaks of several epigenomic marks across multiple cell lines. We analyzed data characterized by broad peaks (H3K36me3, H3K27me3, and EZH2; Section 5.1) and short peaks (CTCF, H3K27ac, and H3K4me3; Section 5.2). Two isogenic replicates of each cell line were used in the analysis. Using RNA-seq data, we assessed the practical significance of our results by associating the detection and classification of differential combinatorial patterns from called peaks with gene expression (Section 5.3; online Appendix D1).

We compared the genome-wide performance of epigraHMM with ChIPComp, csaw, DiffBind, diffReps, RSEG, and THOR. For two-step DPCs, ChIPComp and DiffBind, we used peak calls from MACS2 as candidate peaks (Zhang et al., 2008). Methods were compared in terms of the coverage of differentially enriched genes, the number and average size of DPCs, the log_2_ fold change (LFC) and Spearman correlation of log_2_ reads counts mapped onto DPCs, the computation time, and maximum memory utilized. Metrics for sensitivity and specificity were defined on the window level and based on the coverage of differentially enriched genes by called peaks (online Appendix D1). Read counts were computed using non-overlapping windows of 500 and 250 bp for broad and short marks, respectively (see online Appendix D2 for a discussion on window size). Results presented here pertain to the analysis of cell lines Helas3 and Hepg2 (see online Appendix D3 for additional results). Analyses were adjusted by input control samples in methods that are designed to do so (ChIPComp, DiffBind, diffReps, and THOR). We did not observe a significant improvement in performance by accounting for input control effect or GC-content bias with epigraHMM and did not adjust our analyses for these effects (online Appendix B1).

### 5.1 | Analysis of ChIP-seq data from broad marks

**FIGURE 3:**
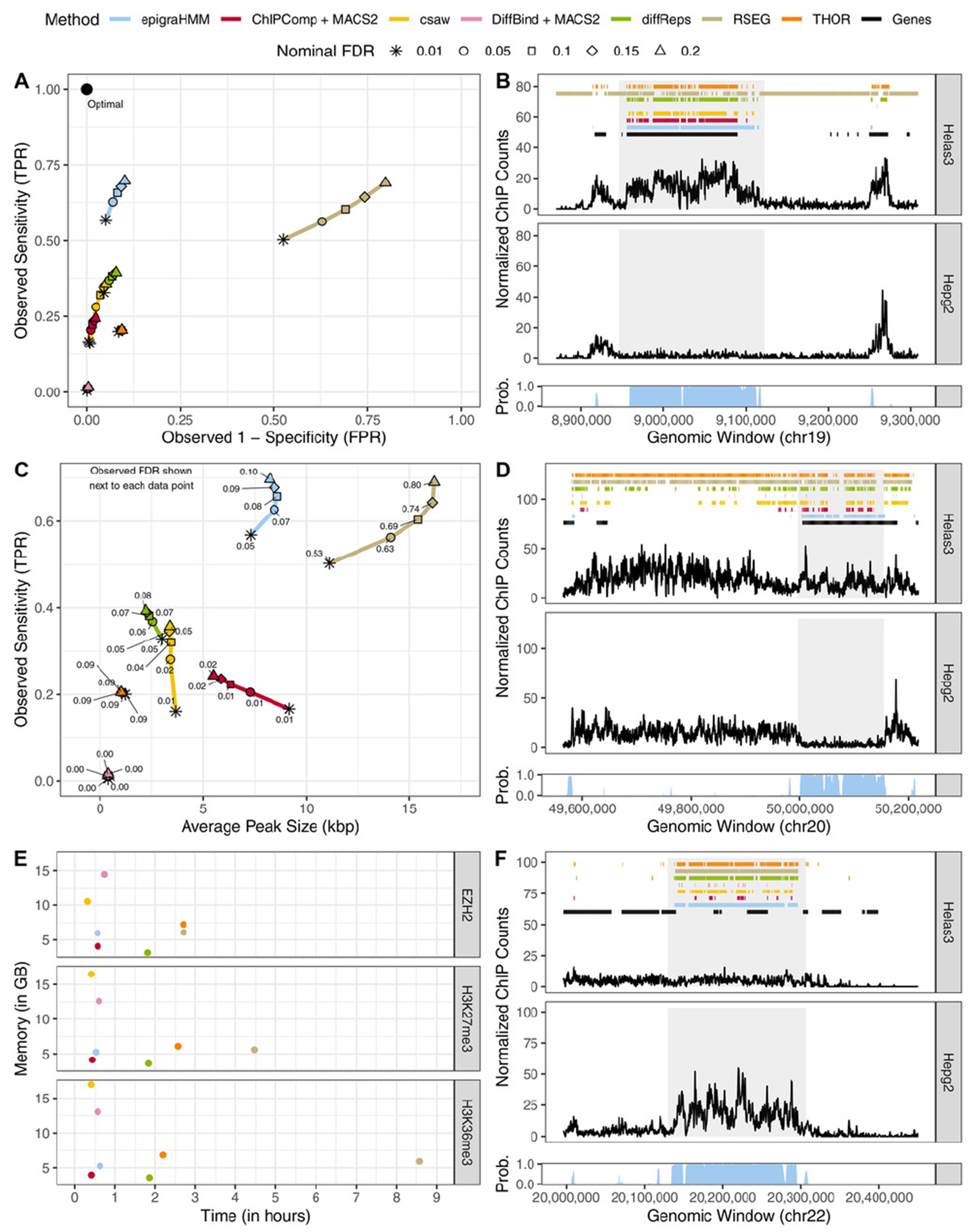
Analysis of broad ENCODE data. (A) ROC curves of DPCs covering differentially enriched genes (|LFC| >2 of ChIP-seq counts) for H3K36me3. (C) Sensitivity (*y*-axis) and average H3K36me3 differential peak size (kbp; *x*-axis) of various methods under different nominal FDR thresholds (observed FDR annotated next to data points). (B, D, F) Example of peak calls from H3K36me3, H3K27me3, and EZH2, respectively, under a nominal FDR control of 0.05. Posterior probabilities of the HMM differential state are shown at the bottom of each panel. Shaded areas highlight differential peak regions. (E) Computing time and peak memory usage of genome-wide analysis from various methods. This figure appears in color in the electronic version of this article, and any mention of color refers to that version.

We compared methods regarding the coverage of differentially enriched genes by DPCs for H3K36me3, an epigenomic mark associated with transcribed genes. Following Ji et al. (2013), in which the authors define true differential binding sites from LFCs of ChIP-seq counts to assess model sensitivity (Figure 2E in Ji et al. (2013)), we defined a set of protein-coding genes exhibiting |LFC| > 2 of ChIP-seq counts between Helas3 and Hepg2 cell lines as true differential binding sites (see online Appendix D1 for results using different thresholding values). Median ratio normalization of counts was performed on protein-coding genes after excluding those with total counts under the 25th percentile of the distribution. Figure 3A shows receiver operating characteristic (ROC) curves for various methods and different nominal FDR levels for H3K36me3 DPCs. Observed true (false) positive rates were computed on the window level as the proportion of windows called as differential out of all windows associated (not associated) with differentially enriched genes (online Appendix D1). Overall, epigraHMM outperformed other DPCs by covering most of the differentially enriched genes while calling a limited number of false positive peaks.

DiffBind, which has been shown to be dependent on the set of candidate peaks (Lun and Smyth, 2015), called the shortest peaks overall (Figure 3B,C) and ChIPComp, which has been shown to perform best in scenarios with short marks (Steinhauser et al., 2016), appeared to have limited performance in broad marks such as H3K27me3 and EZH2 (Figure 3D,F). epigraHMM and RSEG, two HMM-based methods, called broader differential peaks and exhibited better sensitivity than other methods. Yet, differential peaks from RSEG often did not correspond to observed differential enrichment, a fact that explains its high observed FDR and agrees with our simulations (Figures 3C and 2) and others (Starmer and Magnuson, 2016). Our HMM-based approach with a nonlinear normalization via model offsets allowed us to maintain a low observed FDR and a higher sensitivity than other DPCs. Overall, epigraHMM was among the most efficient algorithms due to our computational scheme, taking approximately 30 min to analyze genome-wide data (Figure 3E).

### 5.2 | Analysis of ChIP-seq data from short marks

We further evaluated the performance epigraHMM on ChIP-seq samples characterized by short peaks (CTCF, H3K4me3, and H3K27ac). The goal of our analysis was to assess whether epigraHMM was flexible to different types of data and still able to call short differential regions of enrichment. Here, differential peaks are usually observed in isolated genomic regions and exhibit a high SNR. It has been shown that certain HMM-based algorithms, including RSEG, have low accuracy with such short marks (Hocking et al., 2016).

**FIGURE 4:**
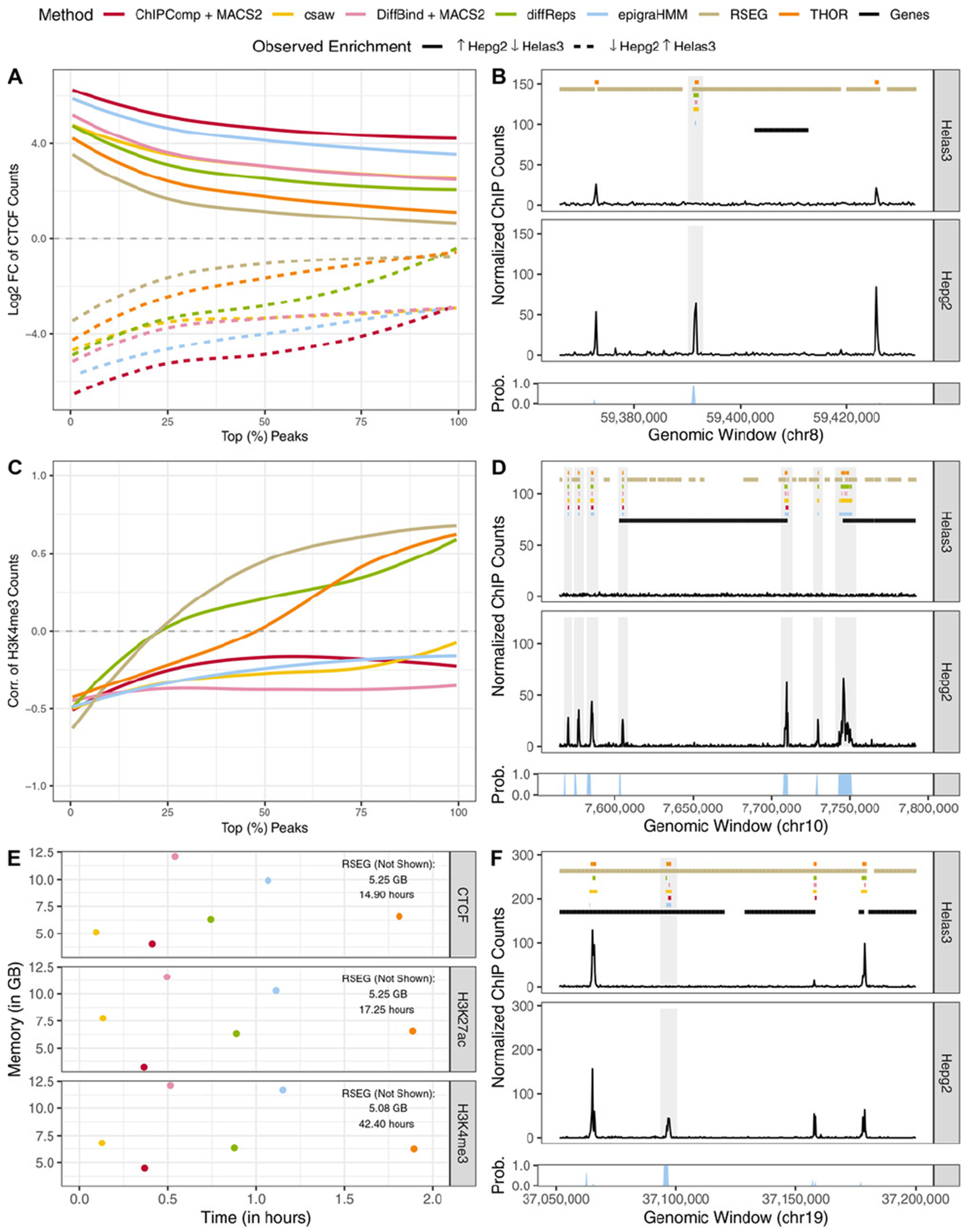
Analysis of short ENCODE data. (A, C): Median LFC and correlation between cell lines of ChIP-seq counts from differential peaks for CTCF and H3K4me3, respectively. (B, D, F) Example of peak calls from CTCF, H3K4me3, and H3K27ac, respectively. Posterior probabilities of the HMM differential state are shown at the bottom of each panel. Shaded areas highlight differential peak regions. (E) Computing time and peak memory usage of genome-wide analysis from various methods. Results are shown under a nominal FDR control of 0.05. This figure appears in color in the electronic version of this article, and any mention of color refers to that version.

We calculated the LFC and the Spearman correlation between cell lines Helas3 and Hepg2 of ChIP-seq counts mapped onto differential peaks called by each method. Median ratio normalization of counts was performed prior to computing such metrics. Here, ideal methods would show high absolute LFC and negative correlation between read counts mapped onto their differential peaks. We observed that other HMM-based algorithms, RSEG and THOR, were among those with the lowest absolute LFC among all methods, which confirms their sub optimal performance in the scenario of short peaks (Figure 4A,C). ChIPComp, DiffBind, and csaw, were among those with the best performance overall, as their DPCs had the highest absolute LFCs and the lowest Spearman correlation of counts between cell lines. Overall, epigraHMM performed comparably to these methods that are known to perform best in short epigenomic marks while calling differential peaks in less than 1.5 hour (Figure 4B,D–F; Steinhauser et al. (2016)).

### 5.3 | Genomic segmentation and classification of chromatin states

We analyzed data from the cell line Helas3 to segment its genome regarding the joint activity of H3K36me3, H3K27me3, and EZH2. We considered each mark as a separate experimental condition (*G* = 3) and jointly classified local chromatin states based upon the presence or absence of enrichment from each mark. Assuming that EZH2 catalyzes the methylation of H3K27me3, a repressive mark, and H3K36me3 associates with transcribed genes (Section 2), we expected consensus enrichment regions to be rare and differential regions to be mostly represented by either transcribed chromatin states (enrichment of H3K36me3 alone) or repressed chromatin states (co-enrichment for H3K27me3 and EZH2). The analyses presented here highlight the applicability of our method in the context of genomic segmentation (Ernst and Kellis, 2012), a distinct problem not tackled by current DPCs.

**FIGURE 5:**
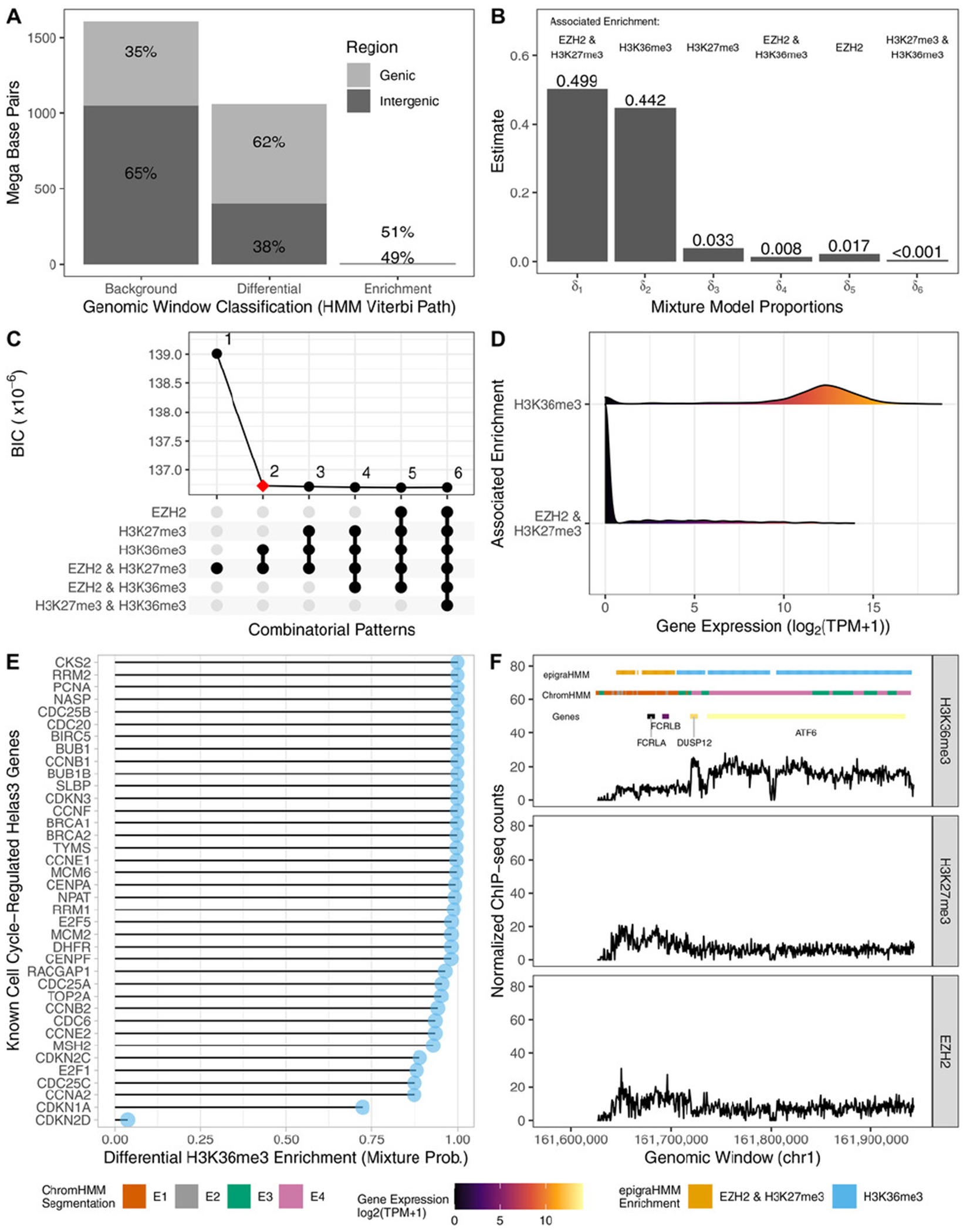
Genomic chromatin state segmentation and classification. (A) Distribution of base pairs (*y*-axis) and the Viterbi sequence of states (*x*-axis). (B) Estimated mixture probabilities and the associated differential combinatorial patterns. (C) Choice of best model with two mixture components via BIC. (D) Density estimate from expression of genes intersecting differential peaks associated with the enrichment of H3K36me3 alone or the enrichment of H3K27me3 and EZH2 in consensus. (E) Cell cycle-regulated genes and their associated average mixture posterior probabilities associated with differential H3K36me3 enrichment pattern of overlapping differential peaks. (F) Example of a genomic region with differential peaks and genes, colored according to their classification and expression levels, respectively. This figure appears in color in the electronic version of this article, and any mention of color refers to that version.

First, we observed that while the majority of genomic regions exhibited no enrichment for any of the analyzed marks, regions of consensus enrichment were rare and covered only 2% of the genome (Figure 5A), as expected. Consensus background and differential regions mostly covered intergenic and protein-coding genic regions, respectively. In fact, differential genomic windows were mostly representing either transcribed chromatin states or repressed chromatin states (Figure 5B). All differential combinatorial patterns expected to be rare had estimated mixture model proportions less than 0.05, which were then pruned using a model selection scheme via BIC (Figure 5C; online Appendix B2).

To assess the biological significance of our results, we associated the distribution of transcripts per million (TPM, from matching RNA-seq experiments) of protein-coding genes with combinatorial patterns of overlapping differential peaks. To assign combinatorial patterns to differential peaks, we chose the combination pertaining to the most prevalent mixture component across windows by using the maximum estimated mixture model posterior probability, $Pr\left(W_{jl}=1|Z_{j}=2,y_{..j},x;\Psi^{\left(t\right)}\right),j=1,\ldots ,M$. Genes associated with transcribed chromatin states had a significantly higher distribution of TPM counts than genes associated with transcribed chromatin states had a significantly higher distribution of TPM counts than genes associated with repressed chromatin states (Figure 5D). In fact, nearly all known Helas3 cell cycle-regulated genes (therefore genes expressed at some stage of the cell cycle; Whitfield et al. (2002)) were associated with high average mixture posterior probabilities of differential enrichment for H3K36me3 alone (Figure 5E).

The expression of such genes are known to correlate with proliferative states of tumors and their study may help characterize their role in cancer (Whitfield et al., 2002). Differential peaks called by epigraHMM, and their assigned differential combinatorial pattern, often agreed with the biological roles as well as the expression levels of associated genes (Figure 5F; results from ChromHMM with four states for comparison). epigraHMM offers the benefit of simultaneously detecting differential peaks and classifying differential combinatorial patterns of enrichment even in the context of genomic segmentation of highly diverse epigenomic marks (online Appendix D4). By using the BIC for model selection, one can choose the number of biologically relevant mixture components to be included in the model, a task that may not be as straightforward in methods such as ChromHMM (online Appendix B2).

## 6 | DISCUSSION

We presented a flexible and efficient statistical model designed to call differential regions of enrichment from ChIP-seq experiments with multiple replicates and multiple conditions. Our model has three main advantages over current methods tailored for differential peak detection. First, it uses an HMM-based approach that accounts for the local dependency of ChIP-seq counts and is able to precisely detect broad and short differential regions of enrichment. Second, it utilizes a GLM-based framework with model offsets that accounts for nonlinear biases and other potential factors such as GC-content bias or input control samples. Our efficient implementation of the RCEM algorithm led to genome-wide analyses of ChIP-seq data under a computational time comparable to some of the fastest current algorithms. Our results highlight the importance of the inclusion of technical/biological replicates in the analysis of epigenomic data. Lastly, our method allows the simultaneous detection and classification of differential combinatorial patterns of enrichment from its embedded mixture model and the associated posterior probabilities under any number of conditions. When certain differential combinatorial patterns are rare, our model selection scheme via BIC provides means of pruning specific patterns of interest (online Appendix B2).

## Supplementary Material

Supplementary Material

Web Appendices A–D, referenced in Sections 1–6, and a link to the implemented software are available with this paper at the Biometrics website on Wiley Online Library. MACS2 peak caller is available at https://github.com/macs3-project/MACS. epigraHMM has been implemented as an R package and it is available for download at https://github.com/plbaldoni/epigraHMM. All of the scripts used in the analyses presented in this paper can be downloaded at https://github.com/plbaldoni/epigraHMMPaper.

## Figures and Tables

**TABLE 1:** Read count simulation. True values and average relative bias of parameter estimates (and 2.5th, 97.5th percentiles) across 100 simulated data sets are shown for H3K27me3 data with 10^6^ genomic windows and ENCODE-estimated SNR

Conditions	Parameter	True value	One replicate		Two replicates		Four replicates	
			Relative bias	(*P*_2.5_, *P*_97.5_)	Relative bias	(*P*_2.5_, *P*_97.5_)	Relative bias	(*P*_2.5_, *P*_97.5_)
Two	*β*_1_	1.116	0.000	(−0.001, 0.001)	0.000	(−0.001, 0.001)	0.000	(−0.001, 0.001)
	*β*_3_	1.165	0.000	(−0.002, 0.001)	0.000	(−0.001, 0.001)	0.000	(−0.001, 0.001)
	*λ*_1_	1.281	0.000	(−0.004, 0.004)	0.000	(−0.003, 0.003)	0.000	(−0.002, 0.002)
	*λ*_3_	0.124	0.004	(−0.057, 0.066)	−0.004	(−0.047, 0.038)	−0.003	(−0.035, 0.034)

Three	*β*_1_	1.116	−0.003	(−0.005, −0.002)	0.000	(−0.001, 0.000)	0.000	(−0.001, 0.001)
	*β*_3_	1.165	−0.007	(−0.010, −0.005)	0.000	(−0.001, 0.001)	0.000	(−0.001, 0.001)
	*λ*_1_	1.281	−0.001	(−0.006, 0.003)	0.001	(−0.002, 0.004)	0.000	(−0.002, 0.002)
	*λ*_3_	0.124	−0.317	(−0.404, −0.223)	−0.023	(−0.057, 0.012)	−0.003	(−0.025, 0.024)

Four	*β*_1_	1.116	−0.014	(−0.015, −0.012)	−0.007	(−0.008, −0.006)	0.000	(−0.001, 0.000)
	*β*_3_	1.165	−0.111	(−0.114, −0.109)	−0.003	(−0.004, −0.002)	0.000	(−0.001, 0.001)
	*λ*_1_	1.281	−0.012	(−0.016, −0.007)	0.007	(0.004, 0.010)	0.000	(−0.001, 0.002)
	*λ*_3_	0.124	−3.520	(−3.589, −3.439)	−0.360	(−0.446, −0.282)	−0.005	(−0.025, 0.017)

## DATA AVALABILITY STATEMENT

The data that support the findings of this paper are openly available in the Encyclopedia of DNA Elements Project portal at https://www.encodeproject.org (Sloan et al., 2016).
